# Label-Free Optical Detection of Pathogenic Bacteria and Fungi at Extremely Low Cell Densities for Rapid Antibiotic Susceptibility Testing

**DOI:** 10.3389/fbioe.2022.884200

**Published:** 2022-06-30

**Authors:** Michael Farid, Marinelle Rodrigues, Robert England, Erdal Toprak

**Affiliations:** ^1^ Department of Pharmacology, University of Texas Southwestern Medical Center, Dallas, TX, United States; ^2^ Independent Engineering and Technology Consultant, Dallas, TX, United States; ^3^ Lyda Hill Department of Bioinformatics, University of Texas Southwestern Medical Center, Dallas, TX, United States

**Keywords:** AST (antibiotic susceptibility testing), optical fiber, biomedical application, clinical microbial infection, rapid assay

## Abstract

Antibiotic resistance is a rapidly expanding public health problem across the globe leading to prolonged hospital admissions, increased morbidity and mortality, and associated high healthcare costs. Effective treatment of bacterial infections requires timely and correct antibiotic administration to the patients which relies on rapid phenotyping of disease-causing bacteria. Currently, antibiotic susceptibility tests can take several days and as a result, indiscriminate antibiotic use has exacerbated the evolution and spread of antibiotic resistance in clinical and community settings. In order to address this problem, we have developed a novel optical apparatus that we called RUSD (Rapid Ultra-Sensitive Detection). RUSD is built around a hollow silica fiber and utilizes bacterial cells as spatial light modulators. This generates a highly sensitive modulation transfer function due to the narrow reflectivity angle in the fiber-media interface. We leveraged the RUSD technology to allow for robust bacterial and fungal detection. RUSD can now detect pathogenic cell densities in a large dynamic window (OD_600_ from ∼10^−7^ to 10^−1^). Finally, we can generate dose response curves for various pathogens and antimicrobial compounds within one to three hours by using RUSD. Our antibiotic- susceptibility testing (AST) assay that we call iFAST (in-Fiber-Antibiotic-Susceptibility-Testing) is fast, highly sensitive, and does not change the existing workflow in clinical settings as it is compatible with FDA-approved AST. Thus, RUSD platform is a viable tool that will expedite decision-making process in the treatment of infectious diseases and positively impact the antibiotic resistance problem in the long term by minimizing the use of ineffective antibiotics.

## Introduction

Effective clinical diagnoses of infections require rapid and accurate species characterization and antibiotic-susceptibility testing (AST) of disease agents to develop most efficacious treatment strategies (Singh et al., 2006; van Belkum et al., 2020). AST in particular is vital due to the ubiquitous nature of antibiotic resistance genes leading to a significant risk of antibiotic treatment failures, particularly for infections occurring in clinical settings (Merli et al., 2015). Such failures not only cause short term problems but can also cause long-term health problems because antibiotics can induce dysbiosis in patient microbiome (Francino 2016).

Current methods to determine antibiotic susceptibility testing of clinical isolates mostly use culture-based techniques that rely on visual inspection or optical density-based measurements for final result (van Belkum et al., 2020). These methods are time-consuming, taking up to several days to obtain results in a setting where prompt results could play a deterministic role in patient health outcomes. A key disadvantage to current optical density-based diagnostics is decreased confidence at low values of density or slow growing pathogens. Consequentially a reliable reading for growth measured by spectrophotometry can only be attained for *Escherichia coli* when there are 10^6^ cells/ml (approx. OD_600_ 0.01) (Cansizoglu et al., 2019). Therefore, these assays are limited by the variation in growth rates of different pathogenic species so the time taken to arrive at these high cell densities could be considerable. Instead, a method able to detect the presence and subsequent growth of even small numbers of bacteria would greatly accelerate current diagnostics.

**FIGURE 1:**
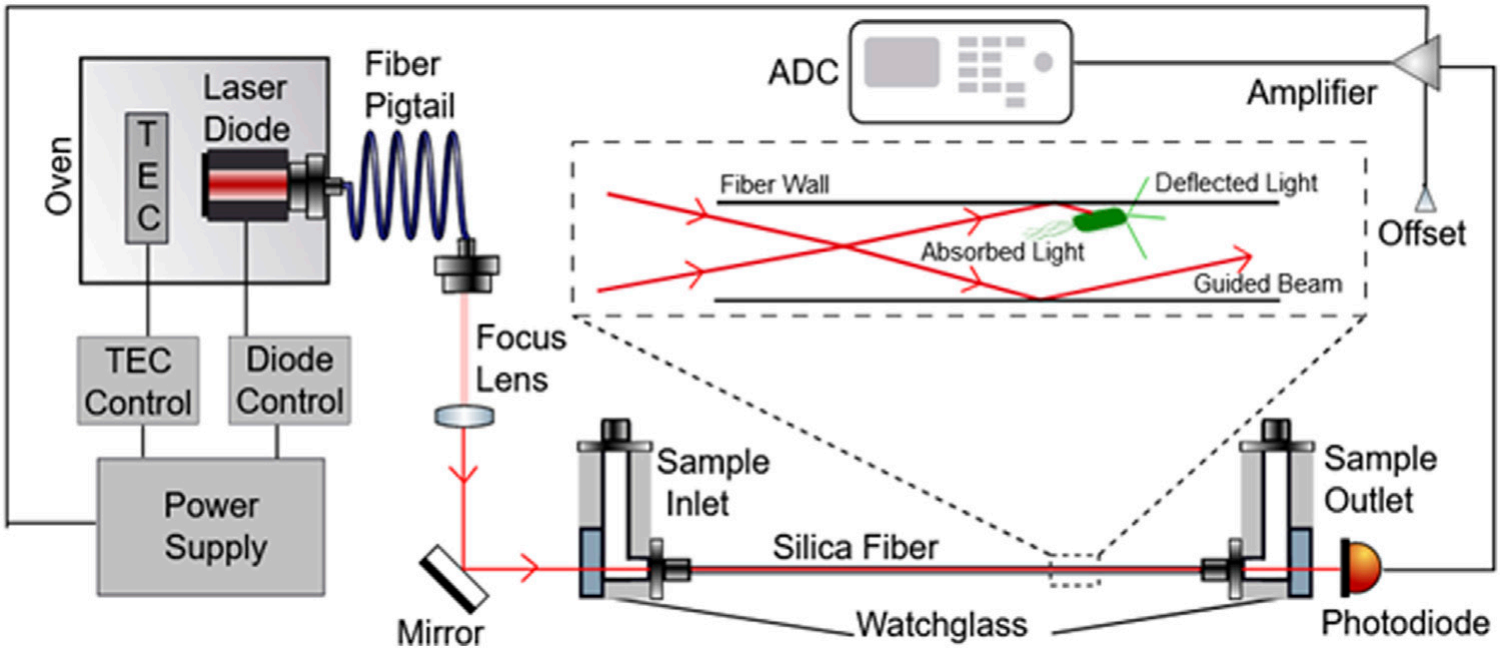
Schematic depicting the RUSD device components. The laser beam emitted by the laser diode is directed into the optical fiber through a focus lens and mirror such that the angle at which the beam enters the fiber is >89°. The Sample inlet part of the fiber also is fitted with tubing used to inject fluid samples into the fiber as well. On the other end of the fiber, the sample outlet directs the liquid sample out of the tube. A photodiode is also present at this end of the fiber to measure the optical signal received from light that was able to pass completely through the optical fiber and convert these units into voltage units. This diode is connected to an amplifier that feeds signal into an ADC which converts these signals into a digital output.

We have previously described a novel experimental apparatus [Rapid Ultrasensitive Detector (RUSD)] utilizing scattering of light as a method for detecting small numbers of bacteria in culture (Cansizoglu et al., 2019). We have validated our findings by showing faster, more accurate results for detecting small numbers of prokaryotic bacterial cells and small numbers of eukaryotic yeast cells. The RUSD is capable of detecting as small as single pathogenic fungal cells such as *Candida glabrata* and ∼25 bacterial cells (e.g., *E. coli*) in its detection volume (∼80 μl). The set-up utilizes a hollow fiber which serves as an opto-fluidic channel and a light guide. Media is pumped through this fiber continuously with the use of programmable pumps or can be injected manually. A laser is focused through the fiber in such a way that the incidence angle at the fiber wall is >89° (almost parallel to the long axis of the fiber), thus ensuring light is total internally reflected through the fiber (Figure 1). Photons that encounter micron sized particles in the media get deflected and can no longer travel through the fiber because they violate the condition for reflectivity. Thus, light encountering obstacles in their path will be blocked and will result in a decrease of detected light intensity at the other end of the fiber (Figure 1). This reduction in light intensity relative to media that is free of obstacles (e.g., bacterial cells) can be measured by the change in voltage captured by a photodiode (hereafter referred to as ΔV). These differences can be converted to OD_600_ values (i.e., OD_600_ = 1 corresponds to approximately 5 × 10^8^ cells/ml of *E. coli* culture).

**FIGURE 2:**
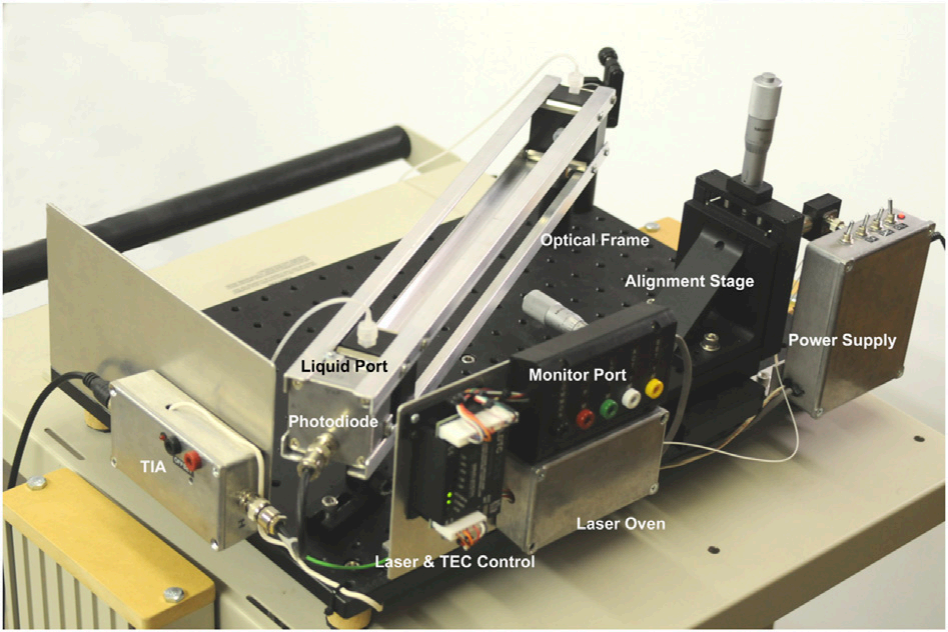
Image of the RUSD device. The image is labelled with the salient features of the device. TIA, trans Impedance amplifier; TEC, thermo electric cooler.

In this paper, we introduce an instrument built with RUSD specifications which is portable, cost-effective and performs at comparable analytical power with adjustable dynamical range for cell density measurements (Figures 1, 2). The device is equipped with a hollow fiber mounted for stability and the laser-photo diode setup for detection of cells. There are some small differences between the previously developed RUSD apparatus and the instrument we describe in this paper. Besides including a temperature control module for the laser power stability, the RUSD device was built with clinical and field use in mind, and as such was constructed in a more compact form. Additionally, the electronic and mechanical components of the RUSD device were redesigned to ensure ease of use, robustness, and simplicity. A detailed schematic describing the components of the device can be found in Figure 1. We show the limit of detection (LOD) of this device is 1,000–10,000 times lower than the LOD for conventional spectrophotometers and plate readers. The adjustable dynamic range of detectable densities allows us to quantitatively measure phenotypic signatures of pathogens such as growth rates much faster than conventional optical-density-based methods. Finally, we use this new RUSD prototype to determine the MIC of a clinically relevant antibiotic, cefepime, arriving at a conclusive result for inhibitory/sub-inhibitory concentrations in 1 h versus at least 8–10 h using conventional methods.

## Results

### Range of Detection

**FIGURE 3:**
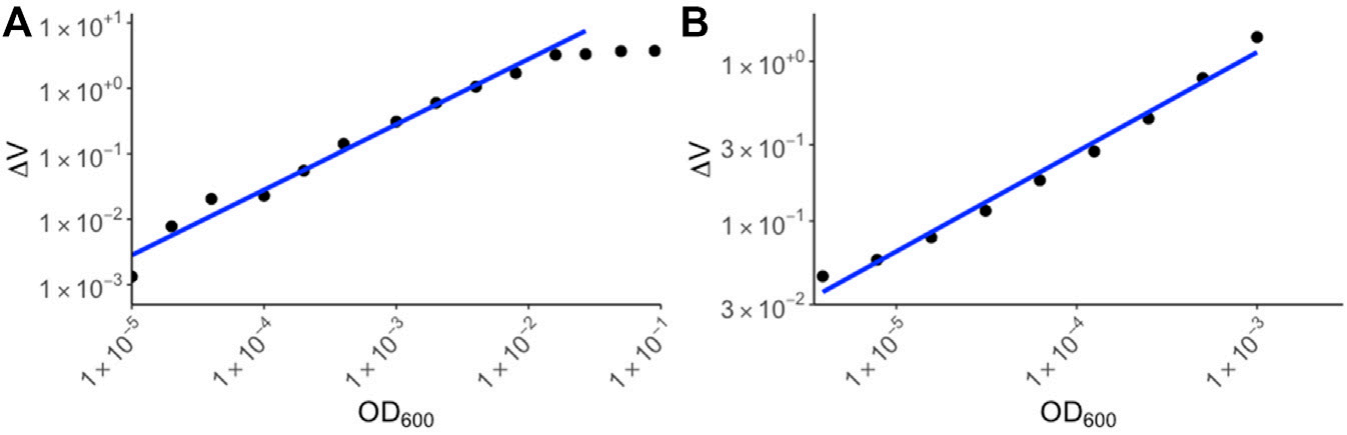
Calibration curves for yeast and *E. coli*. **(A)** *E. coli* BW25113 strains were cultured overnight in M9 media and the following day, serial dilutions were made in M9 media supplemented with chloramphenicol. The ΔV values were plotted against OD_600_ values corresponding to spectrophotometer values of the original culture. **(B)** Yeast strain *S. cerevisiae* was cultured in YPD media and dilutions of the culture were made. ΔV values were measured using RUSD device and were plotted against spectrophotometer values based on dilutions of the overnight culture.

To determine the range of detection for the device, overnight *E. coli* cultures were serially diluted and the ΔV for each of the diluted samples were measured using RUSD (Figure 3A). The ODs were calculated using a spectrophotometer and corresponding values for colony forming units (or CFUs) were also simultaneously determined by colony counting method. For our calibration we started at OD_600_ ∼3 × 10^−6^ corresponding to 1,500 CFU/ml (Figure 3A). This represents a ∼1,000 -fold more sensitivity compared to conventional spectrophotometers whose lower limit of detection (LOD) typically range between 10^−3^ and 10^−2^.

The maximum OD_600_ that could be detected using the RUSD setting we used for this paper was ∼10^−2^ corresponding to 5 × 10^6^ bacterial cells per milliliter. At these cell densities the instrument reached its maximum reading threshold and higher densities of cells registered identical saturated readings (Figure 3A). This metric shows the sensitivity of the RUSD compared to conventional optical-based methods. While spectrophotometers often have a range of detection spanning 10^6^ CFU/ml–5 × 10^8^ CFU/ml, the range for the RUSD is much wider for far lower cell densities leading to more precise detection of lower quantities of cells in biological samples. M9 minimal growth media was used in the device due to its transparent color enabling more sensitive detection of smaller numbers of cells (Methods). Of note, the dynamic range can be rapidly adjusted on the fly and the same RUSD device can perform equally well at different dynamical ranges when necessary.

Eukaryotic cells such as yeast are generally larger than bacteria and hence their detection range differs. Using yeast cultures in YPD media, we made a calibration curve starting at OD_600_ 10^−6^. (∼20 cells) to OD_600_ 10^−3^ (Figure 3B). The sensitivity of the RUSD can be increased when necessary to detect even single yeast cells by simply increasing laser power and gain in the detector.

### RUSD for Detecting Cell Growth Dynamics

**FIGURE 4:**
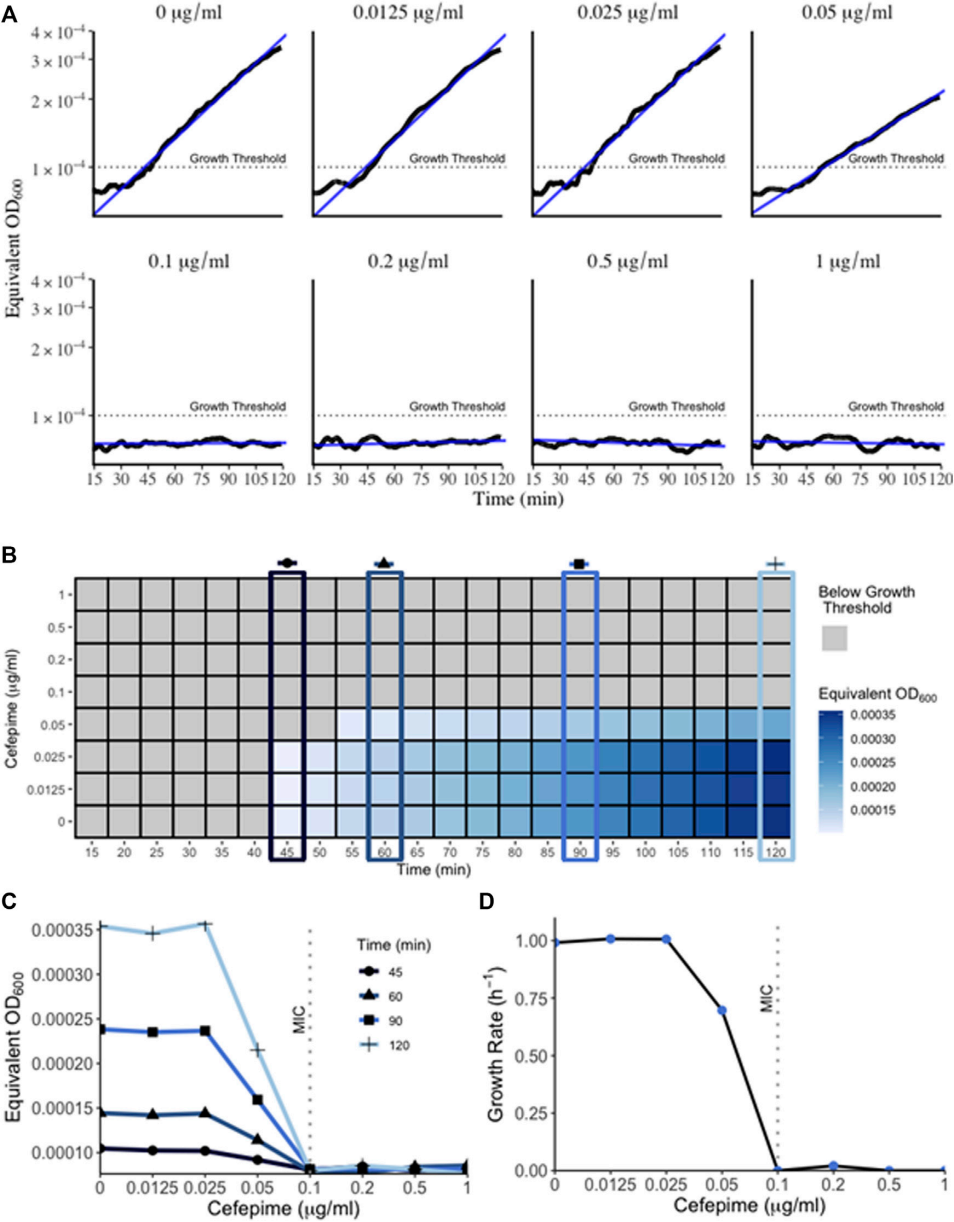
Growth of bacteria in RUSD device. **(A)** Overnight cultures of *E. coli* were diluted to 5 × 10^−5^ OD_600_ and circulated through the device for 2 h in M9 media supplemented with different concentrations of cefepime. Bacterial growth can be detected as early as 45 min. Growth within the 2-h time frame indicated sub-MIC concentrations of cefepime. **(B)** Heat map of bacterial OD_600_ (equivalent of measured as RUSD ΔV) versus different concentrations of cefepime over time. T = 45, 60, 90, and 120 min are highlighted to emphasize the earliest interpretation of antibiotic resistance/susceptibility. **(C)** Plot of equivalent OD_600_ of cultures at different times during growth with cefepime. Definitive results for growth (i.e., resistance/susceptibility) can be seen after only 1 h. **(D)** Figure showing growth rate vs. concentration of cefepime as measured in the RUSD device.

The bacterial “lag” phase refers to the initial period when bacteria are introduced into a new environment when no growth is detectable (Bertrand 2019). When measured with conventional methods such as a plate reader or spectrophotometer this phase can last several hours depending on the initial density of the inoculum. However, one reason for this extended lag time is because these methods can only detect higher cell densities. Bacterial growth below the method’s limit of detection is not detected and falsely extends the observed lag time (Cansizoglu et al., 2019). Therefore, methods that can measure miniscule concentrations of bacteria at that initial growth phase present a method to combat inflated lag times delaying results. Using a very low concentration initial inoculum of 5 × 10^−5^ OD_600_ (approx. 25,000 CFU/ml) in the RUSD, we were able to detect growth in as little as 45 min (Figure 4) following inoculation. This represents an important technical advancement since measuring growth of a culture is an essential step when determining Minimum Inhibitory Concentrations (MIC) for antibiotics and performing antibiotic susceptibility testing (AST).

We followed the growth curve of this inoculum using an initial density of 5 × 10^−5^ and were able to obtain a growth curve that matched the growth dynamics such as growth rate and doubling time (∼40 min in minimal M9 media, Figure 4D) obtained using other optical methods at higher cell densities (Reshes et al., 2008). This illustrates how the RUSD device can replicate bacterial growth measurements obtained from other methods even for cultures with a much lower initial bacterial concentration, which attests to the accuracy of this device at lower cell densities.

### Rapid Antibiotic Susceptibility Testing With iFAST

Timely determination of antibiotic susceptibilities and MIC is important in clinical settings to decide effective treatment interventions. However, since these clinical AST assays are usually based on spectrophotometer readings and rely on substantial bacterial growth, these can take up to several days to obtain a result. Using the RUSD, we have established a novel technique to quickly determine the MIC of a sample termed “iFAST” or in-Fiber-Antibiotic-Susceptibility-Testing. In this method, using the RUSD and bacterial inoculums of 5 × 10^−5^ OD, we determined the MIC of *E. coli* to the antibiotic cefepime (a fourth-generation cephalosporin). Since our current set up has a single fiber, we were only able to use one antibiotic concentration at a time, but each concentration gave us definitive readings for growth and growth rate by hour two post injection and using these values, we were able to determine the MIC for cefepime within 45 min (Figure 4B) hours, substantially faster than any other AST method known to us. The first 15 min of data measurements were not shown in Figure 4B as the readings from the device were erroneous due to the bacteria still mixing in the fiber after injection. Using these values, we were able to plot the equivalent OD_600_ of cultures measured as ΔV in the RUSD versus time for the different antibiotic concentrations used (Figure 4C). The graphs show the magnitude of the difference between the different antibiotic concentrations which becomes visible as early as 45 min after the start of the assay and progresses for the duration of the 2 h.

## Conclusion and Discussion

In conclusion, our the RUSD device we developed is able to recapitulate the benefits of our previously validated experimental setup with small reductions in sensitivity in exchange for greater stability and adjustable dynamic range. The lower limit of detection for this instrument is key to its advantage over other conventional methods since it can detect fewer cells in the medium and monitor their growth very early. Another advantage of this setup is how it fits in with standard operating procedures already in place for clinical AST.

Currently in clinics, blood cultures are the gold standard for the diagnosis of sepsis and bacteremia, despite the test requiring many hours or even days to return a result leading to prolonged treatment using empiric broad-spectrum antibiotics (Sweeney et al., 2019). A positive blood culture, while indicative of an underlying infection does not yield any information on species identification and antibiotic susceptibility testing. These types of tests are often performed using matrix-assisted laser desorption ionization time-of-flight (_MALDI-TOF) or nucleic-acid-based methods (PCR or sequencing) (Opota et al., 2015) which can add hours to the proper diagnosis of the underlying cause of infection worsening patient outcomes. AST testing which relies on growth-based assays is also a time-consuming step relying on standard broth microdilution assays or disk diffusion assays (CLSI, 2012; CLSI, 2018).

There is currently an urgent need to develop AST devices/protocols that are cost-effective and deliver results fast. Some innovative methods that have been developed include lab-on-a-chip setups that use DNA hybridization and amplification (Kalsi et al., 2015). Another promising method uses electrochemical signals from a redox reaction to indicate growth with a particular antibiotic (Besant et al., 2015). These methods, while effective, still require the use of consumable reagents and additional processing steps before the assays can be performed. Our RUSD device, however, is compatible with current clinical lab protocols and will require minimal specialized training to operate. To our knowledge, this is the first application of an optical fiber wherein it is directly used for culturing and detection of bacteria. Other medical devices have employed the use of these fibers merely to transmit signals from a separate detection apparatus (Hayman 2008; Ahmed et al., 2014; Yoo and Lee 2016; Janik et al., 2021). Therefore this technology represents a paradigm shift for clinical laboratories. Using RUSD can save precious hours in clinical testing and enable healthcare workers to quickly proceed with correct treatment of patients thereby alleviating infectious burdens faster. We are currently working towards making RUSD a fully automated and multiplexed platform as antibiotic susceptibility testing require testing many antibiotic compounds at various concentrations. This can be achieved by either building many RUSD devices operating in in parallel or utilizing an advanced fluidic platform for time-sharing between channels and automating sample injection and sterilization.

## Materials and Methods

### Device Design

The core design of the second version of the device is identical to the one described in the original RUSD paper (Cansizoglu et al., 2019). However, several additions were added to the system to ameliorate it (Figures 1, 2). First, the laser diode was placed under temperature control using a closed feed-back loop system to enhance beam stability and quality. The silica fiber we use is highly sensitive to temperature changes because of its large length (e.g., 40 cm) and small wall thickness (∼0.15 mm). This presented an issue when performing susceptibility testing experiments as the higher temperature of the media and incubation environment would alter the power output of the laser diode, thereby skewing the readings. Additionally, a programmable hot plate and a peristaltic pump were added to the system to allow for continuous measurements and growth of bacteria directly in the testing chamber. This addition was invaluable from a practical and user case perspective as it allowed autonomous continuous measurement of bacterial growth rate without the need for taking samples out of the incubator to inject them into the device for measurement. Overall, while the changes might seem minor, they truly expanded the capabilities and robustness of the device as a clinical tool.

### Calibration

Before utilizing the device for AST, we first calibrated the device to have a correspondence formula between voltage drop in the device as a result of the presence of bacteria and their concentration in OD_600_. This was done with *E. coli* bacteria (K12, BW25113) and brewer yeast (*Saccharomyces cerevisiae*). *E. coli* was grown overnight in M9 minimal media supplemented with 0.4% glucose (Fisher Scientific B152-1), 0.2% Amicase (MP Biomedicals 104778), 2 mM MgSO_4_ (Fisher Scientific M63-500), 100 µM of CaCl_2_ (Fisher Scientific S25222A), while yeast was grown in YPD media (Fisher Scientific BP-2469). The following day (or 2 days in the case of yeast) the concentration of the overnight culture was measured using a spectrophotometer and 2-fold dilutions were made in the range between 10^−5^ and 10^−1^ OD for bacteria and 10^−6^ and 10^−3^ for yeast. Then, the voltage drop for each dilution of *E. coli* or yeast was measured for 30 s at 1000 Hz sampling frequency, and the average value of the signal was calculated. Then, log-log linear regression was used to derive the conversion formula for both yeast and *E. coli*.

### Antibiotic Susceptibility Testing

To demonstrate the clinical potential of the new version RUSD, we utilized it to conduct rapid antibiotic susceptibility testing against cefepime, a new-generation beta-lactam antibiotic. Eight culture media solutions (using M9 minimal media supplemented with glucose and cas amino acids) were made with increasing cefepime concentrations ranging from 0 to 1 μg/ml. For each sample, bacteria was added to roughly a level of 0.00005 OD_600_ and then the sample was circulated in the device at 37°C, and measurements were collected using DASYLab^®^ software for 2 h. This was accomplished by placing the sample tube in a hotplate set to 37°C and using a peristaltic pump for pushing the sample solution into the device’s fiber and back to the conical tube. The initial 10–15 min of measurement data was not used as the bacteria was still being mixed in the device chamber, yielding sporadic noisy readings. Between sample measurements, the device was cleaned of any residue by pumping the sample media out of the fiber and running 70% isopropyl alcohol for 2 min. After all eight samples were run through the device and measurements were collected, they were converted to OD_600_ from ΔV and the growth dynamics and MIC could be interpreted starting around 45 min (Figure 4).

## Data Availability

The raw data supporting the conclusion of this article will be made available by the authors, without undue reservation.
